# Clozapine toxicity due to a multiple drug interaction: a case report

**DOI:** 10.1186/s13256-015-0547-2

**Published:** 2015-04-02

**Authors:** Giovanna Cadeddu, Arianna Deidda, Maria Erminia Stochino, Nicola Velluti, Caterina Burrai, Maria Del Zompo

**Affiliations:** 1 Section of Neuroscience and Clinical Pharmacology, Department of Biomedical Sciences, University of Cagliari, SP8, Km. 0,700, 09042 Monserrato, CA Italy; 2 Sardinian Regional Center of Pharmacovigilance, Unit of Clinical Pharmacology, AOUCA, “San Giovanni di Dio Hospital”, Via Ospedale 54, 09124 Cagliari, Italy; 3 Center of Mental Health, ASL8, Via Raffaello 5, 09032 Assemini, CA Italy; 4 Psychiatric Unit, ASL 8, “SS. Trinità” Hospital, Via Is Mirrionis 92, 09121 Cagliari, Italy

**Keywords:** Clozapine, Drugs interaction, Pericarditis

## Abstract

**Introduction:**

We report the case of a multiple drug interaction involving clozapine, antifungals and oral contraceptives, which resulted in an increased clozapine plasma level, pericarditis with pericardial effusion and eosinophilia in a young Caucasian woman. These symptoms and signs disappeared a few days after discontinuation of clozapine. At present, we are not aware of reports of clozapine–antifungals interaction, whereas there is only one other case report on the interaction between oral contraceptives and clozapine. The purpose of this case report is to show the risk of potentially serious adverse effects stemming from drug interactions involving medications routinely used in clinical practice.

**Case presentation:**

A 29-year-old Caucasian woman diagnosed with a schizoaffective disorder was admitted to a psychiatric unit for acute psychosis (hallucinations, delusions and catatonic behavior). She denied smoking tobacco products and was on long-term oral contraceptives. During the first month of hospitalization she was treated with antipsychotics and for 1 week she took simultaneously fluconazole and miconazole gel, after being diagnosed with oral candidiasis. On the last day of antifungals treatment, 29 days after admission, clozapine was started with resolution of psychotic symptoms. After 3 weeks, her clozapine plasma level had increased to 542ng/mL and eosinophilia was observed. She complained of nausea, vomiting and palpitations; echocardiography showed echocardiographic abnormalities and pericardial effusion. Oral contraceptives were discontinued and after 1 week clozapine was interrupted, with a complete resolution of side effects and pericardial effusion within 4 days.

**Conclusions:**

Clozapine is metabolized by cytochrome P450. The use of inhibitors or other substrates of cytochrome P450, such as antifungals and oral contraceptives, can cause long-lasting interactions and clozapine toxicity. The Naranjo algorithm shows clozapine is a definite cause of pericarditis (score 9) and both clozapine–antifungals and clozapine–contraceptives interactions resulted probable (score 5) in Drug Interaction Probability Scale. A good knowledge on drugs that act as substrates, inhibitors or inducers of cytochrome P450 is mandatory. When those drugs are used in patients taking clozapine, blood level monitoring of clozapine should be recommended, since a lower dose of clozapine might be required to prevent clozapine toxicity.

## Introduction

Clozapine is an atypical antipsychotic, known for life-threatening side effects, such as agranulocytosis and cardiac complications, but also for its role in the treatment of resistant psychoses when other therapies have failed [1].

The most serious cardiac complications caused by clozapine, such as cardiomyopathy, myocarditis and pericarditis, are characterized by shortness of breath, heart palpitations/pains and thoracic pain. In most cases, electrocardiographic changes, pericardial effusion, and nonspecific signs of inflammation are observed. However, only a few cases of pericarditis and pericardial effusion induced by clozapine, even when used at low dosage, are reported in the literature [2].

A review by Wehmeier *et al.* reported 65 cases of myocarditis, 52 cases of cardiomyopathy and only six cases of pericarditis occurring during clozapine treatment [3]. The dose used is a poor predictor of clinical response, and there is little correlation between dose and plasma level, due to individual differences in metabolism, pharmacokinetic differences, gender, age, drug interactions and the smoking of tobacco products.

Antifungal drugs, including fluconazole and miconazole, are widely used in the treatment of systemic candidal infections and mycoses. Multiple drug therapy is a common therapeutic practice and many drug–drug interactions involving metabolic inhibition are reported in the literature. Clozapine is metabolized by the hepatic cytochrome P450 (CYP) microsomal system. The contribution of these isoenzymes to clozapine metabolism differs between individuals, leading to the wide inter-patient variability found in clozapine plasma concentration. The drug is converted to norclozapine by CYP3A4 and 1A2 and to clozapine N-oxide by CYP3A4 [4,5]. However, CYP2C19 is also important at clozapine therapeutic concentration (24%) whereas the contributions of CYP2C9 (12%) and 2D6 (6%) are more modest. CYP1A2 is the most important form at therapeutic concentration (30%), while CYP3A4 plays a more important role at a high concentration (37%) than at therapeutic concentration (22%) [6].

The use of inhibitors or other substrates of P450, such as oral contraceptive (OCs) and antifungals, can cause unfavorable and long-lasting interactions and clozapine toxicity: *in vitro*, miconazole and ketoconazole may cause an inhibition of more than 50% of clozapine metabolism [7].

It has been reported that fluconazole and miconazole competitively inhibit CYP3A4 activities.

In particular, CYP3A4, CYP2C9, CYP2C19 and CYP1A2 are more strongly inhibited by miconazole than fluconazole [8], and this inhibition may last several days, due to antifungals long half-life and wash-out, allowing clozapine accumulation and toxicity. In this way, miconazole and fluconazole may inhibit clozapine metabolism determining an increase and/or prolongation of both therapeutic and adverse effects. At present, we are not aware of reports of clozapine–antifungals interaction; however, drug–drug interactions involving other classes of antipsychotics and antifungals are reported in the literature [9], whereas there is only one other case report on the interaction between OCs and clozapine [10]. The purpose of this case report is to show the risk of potentially serious adverse effects stemming from drug interactions involving medications routinely used in clinical practice. Learning how to predict and monitor drug–drug interactions may help reduce the incidence of clinically significant adverse drug events.

## Case presentation

A 29-year-old Caucasian woman affected by a schizoaffective disorder, treated with haloperidol 2mg per day and olanzapine 10mg per day, was admitted at a Psychiatric Unit for a reacutization of her psychotic symptoms (hallucinations, delusions and catatonic behavior), due to a lack of medications adherence. Her past medical history was characterized by a previous hospitalization for acute psychosis 1 year earlier, incomplete right bundle branch block (RBBB) and ovarian cysts. Her family medical history revealed that her mother had an anxiety disorder and her grandmother had a major depression. She was on long-term OCs, ethinyl estradiol/drospirenone 0.03mg/3mg per day and denied smoking tobacco products and any substance use.

A physical examination showed a temperature of 37.2°C and blood pressure of 150/100mmHg, whereas all the other parameters were within normal range. The results of blood tests and electrocardiography (ECG) were normal, except for RBBB. Her hospitalization lasted 3 months and during the first month she was treated orally with olanzapine 20mg per day and haloperidol 9mg per day for 23 days. On the 23rd day of hospitalization, since a poor response to treatment was observed, antipsychotics were interrupted and aripiprazole 30mg per day was administered for 6 days. In addition, on the same day, she was diagnosed with oral candidiasis and treated simultaneously for a week orally with fluconazole 100mg per day and miconazole oral gel 2% 20mg, two times per day. Since her psychotic symptoms did not seem to improve, 29 days after admission and on the last day of antifungal treatment, aripiprazole was replaced by clozapine.

Clozapine was started at 25mg per day orally and was gradually increased, within 16 days, to 225mg per day with the resolution of psychotic symptoms. After 3 weeks the plasma level of clozapine was 542ng/mL (range 350 to 450ng/mL) and the level of its active metabolite norclozapine was 216ng/mL. Blood tests showed eosinophilia and an increase of C-reactive protein (5.73mg/L). She experienced the first symptoms (nausea, vomiting, palpitations) 5 days before the plasma level of clozapine was measured, while she was being treated with clozapine and OCs. At that point, long-term OCs treatment was discontinued and no other form of contraception was administered to her. She was referred to a cardiologist.

A physical examination showed tachycardia and gallop rhythm and she complained of nausea and vomiting. An ECG revealed sinus tachycardia (135 beats/minute), QTc 0.43 seconds, and S-T segment depression and inversion of T-waves in inferior and lateral leads. An echocardiography showed a small pericardial effusion suggestive of iatrogenic pericarditis.

**Figure 1:**
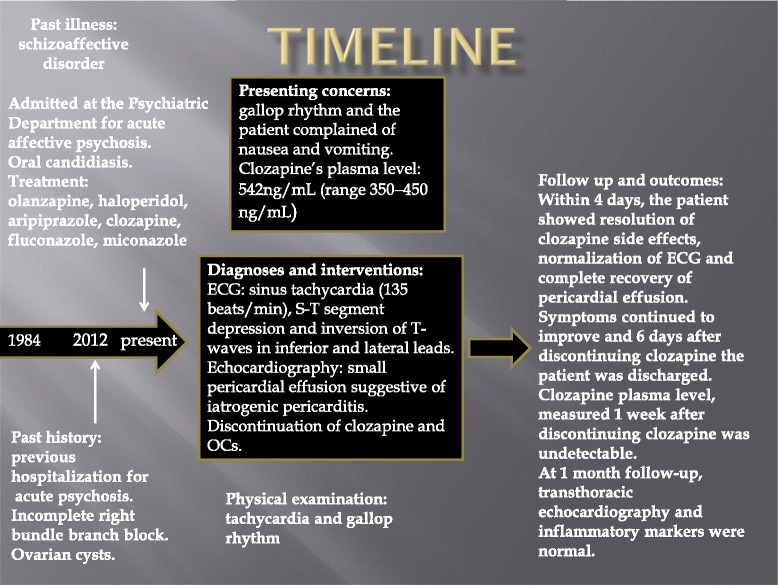
**Clinical case timeline.** The figure reports the most important clinical aspects, interventions, diagnosis and follow up with the outcomes. Abbreviations: ECG, electrocardiography; min, minutes; OCs, oral contraceptives.

Due to those findings, 1 week after discontinuing OCs, clozapine was also interrupted and she was not rechallenged. Within 4 days, she showed resolution of clozapine side effects, normalization of ECG and complete recovery of pericardial effusion. Her symptoms continued to improve and 6 days after discontinuing clozapine she was discharged. The plasma level of clozapine, measured 1 week after discontinuing clozapine (2 weeks after interruption of OCs) was undetectable. At 1 month follow-up, transthoracic echocardiography and inflammatory markers were normal (Figure 1).

## Discussion

We report the case of a multiple drug interaction involving clozapine, OCs and antifungals, which resulted in an increased clozapine plasma level, pericarditis and eosinophilia. Although clozapine toxicity is usually linked with clozapine serum levels higher than 1000ng/mL, at the moment there is no evidence of a clear toxicity range in the literature [11] and we cannot exclude that the patient might have experienced an adverse reaction at a lower serum level of clozapine (542ng/mL). Pericarditis is an insidious disease which may debut with nonspecific symptoms, therefore a high level of diagnostic suspicion is always needed when clozapine is being used. In this case, the most common causes of pericardial illness were ruled out with the possible exception of a viral pericarditis, since cardiac symptoms began 1 month after clozapine was started, quickly resolved after discontinuation and did not return again. Clozapine-induced myocarditis was also excluded since echocardiography, cardiac necrosis markers and brain natriuretic peptide were all negative.

Clozapine was a definite causative agent (score of 9) according to the Naranjo probability scale [12], which evaluates single-drug adverse events, and according to the Drug Interaction Probability Scale (DIPS) [13], a probable causative agent, since both clozapine–antifungals and clozapine–OCs interactions scored 5.

The ranking differences between these two assessment tools involve the questions used in DIPS to evaluate drug–drug interactions; in multiple drug interactions DIPS is the most accurate tool. A positive rechallenge with the precipitant drug is a strong indicator of causation. Unfortunately, the patient was not rechallenged with clozapine, and previous plasma levels of the drug were not measured, so we are not able to rule out the presence of clozapine toxicity prior to the first measurement.

At present, we are not aware of reports of clozapine–antifungals interaction, but drug–drug interactions involving other classes of antipsychotics and antifungals are reported in the literature [9]. 17α-Ethinyl estradiol (EE) is a CYP3A4 substrate and an inhibitor of CYP1A1 *in vitro*; moreover, there is evidence of a time-dependent inhibition of CYP3A4 and CYP3A5 by OCs [14]. Drospirenone is partially metabolized by CYP3A4 and is also an inhibitor of CYP2C19 and CY2C9. Therefore, both OCs’ components can inhibit different CYPs, although at higher clozapine concentrations CYP3A4 is the most active; thus the greater inhibition is more probably determined by EE.

There is less information about the effect of OCs on other drugs, but clinically relevant pharmacokinetic interactions are known to occur. A possible OCs–clozapine interaction mechanism could be a competitive enzyme inhibition, as both drugs are partially metabolized by CYP3A4, and OCs are considered mild inhibitors of CYP3A4. Moreover, several studies have shown that OCs impair the metabolism of CYP1A2 substrates *in vivo* [15], which might represent another possible mechanism (Table 1).

**Table 1:** **Drugs’ metabolism**

**Medication**	**Metabolism**	**Inducers**	**Inhibitors**
**Clozapine**	CYP1A2, 3A4, 2C19, 2C9, 2D6	–	–
**Fluconazole**	Hepatic	–	CYP3A4, 2C9
**Miconazole**	Hepatic	–	CYP3A4, 2C9, 2C19, 1A2
**Ethinyl estradiol**	CYP3A4, 1A2	–	CYP1A2, 2B6, 2C19, 3A4
**Drospirenone**	CYP3A4	–	CYP1A1, 2C9, 2C19

To the best of our knowledge, there is only one report of a possible drug interaction between clozapine (at a dose of 550mg per day) and OCs, in a 47-year-old woman who was a heavy smoker of tobacco products, resulting in elevated clozapine plasma levels (736, 770, and 792ng/mL) and onset of adverse effects, after which OCs treatment was discontinued. Side effects disappeared within a few days and normalization of clozapine blood levels was observed over the next 6 weeks [10].

**Figure 2:**
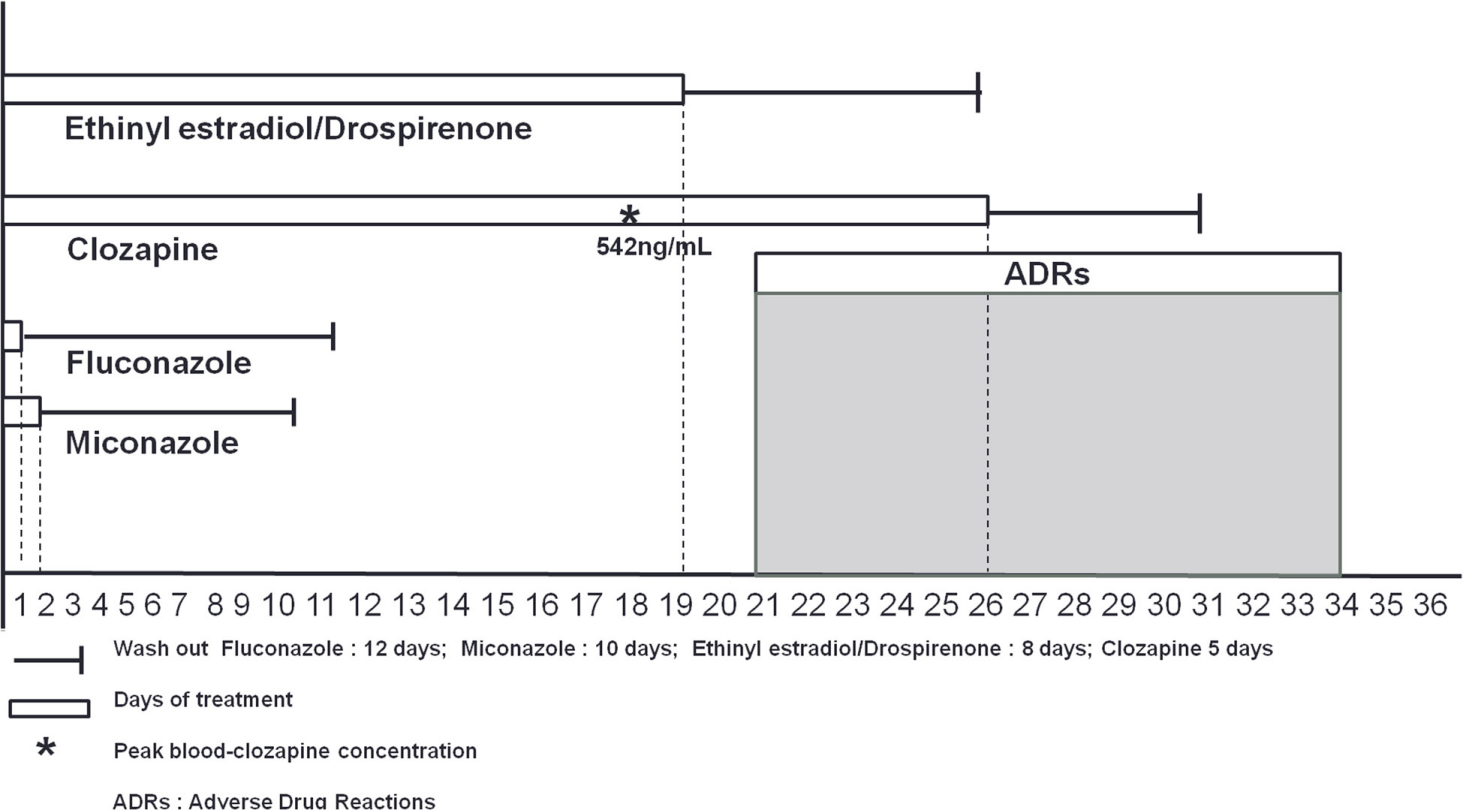
**Drugs’ interaction timeline and pharmacokinetics.** The figure reports the four drugs administered to the patient at the same time (ethinyl estradiol/drospirenone, clozapine, fluconazole, miconazole) and the onset and duration of the adverse drug reactions. The peak of clozapine plasma level (542ng/mL) was observed after 18 days. Adverse drug reactions were observed 2 days after clozapine peak and lasted 14 days. Abbreviation: ADRs, adverse drug reactions.

Finally, the inhibition of CYPs is compatible with the pharmacokinetics of antifungals and OCs, allowing the elevation of clozapine plasma levels which occurred 2 weeks after antifungals interruption. Miconazole and fluconazole half-life and wash-out are 24 hours and 10 days, and 28 hours and 12 days, respectively. In this case, we must take into account not only the single antifungals–clozapine interaction but also the contribution of OCs (half-life 20 hours, wash-out 8 days) which might have impaired CYPs metabolism for 26 days, while the patient was simultaneously treated with clozapine (Figure 2). Since OCs were administered for a longer time, compared to antifungals, we think that they might have contributed more significantly to the increase of the blood concentration of clozapine.

## Conclusions

To conclude, this report suggests a possible multiple drug interaction between clozapine, antifungals and OCs, which resulted in an elevated clozapine blood level, eosinophilia and pericarditis with pericardial effusion.

Based on the case report described here, we can make the following recommendations:

1. In patients taking clozapine, detecting ECG and echocardiography abnormalities is highly recommended, and clozapine must be stopped if patients develop pericardial involvement.
2. Blood level monitoring of clozapine is essential when inhibitors or substrates of CYP3A4 and CYP1A2, such as antifungals or OCs, are being used, since a lower dose of clozapine might be required to prevent adverse effects.
3. A good knowledge of drugs that act as substrates, inhibitors or inducers of P450 is essential for doctors to take appropriate cautions, and a close monitoring for potential drug interactions when using drugs with a narrow therapeutic range such as clozapine is always recommendable.

## Consent

Written informed consent was obtained from the patient for publication of this case report and accompanying images. A copy of the written consent is available for review by the Editor-in-Chief of this journal.

## Abbreviations

CYP: Cytochrome P450
DIPS: Drug Interaction Probability Scale
ECG: Electrocardiography
EE: 17α-Ethinyl estradiol
OCs: Oral contraceptives
RBBB: Right bundle branch block
